# An innovative method of expanding the support for doctors returning to training in psychiatry after a period of extended leave: the Sheffield Mindful Support Programme

**DOI:** 10.1192/bjo.2021.409

**Published:** 2021-06-18

**Authors:** Helen Linnington, Hamid Alhaj

**Affiliations:** Sheffield Health and Social Care NHS Foundation Trust

## Abstract

**Aims:**

To offer regular continuous professional development opportunities covering both clinical and non-clinical skills to trainees and trainers and enhance their experience and skills to increase their wellbeing and resilience.

**Background:**

There are approximately 50,000 doctors undertaking postgraduate training in England. Of these, 10% (5000) are taking approved time out of training at any time. A 2017 HEE survey revealed that doctors returning to work reported numerous concerns. Based on these and with the backdrop of the Bawa-Gaba case HEE's Supported return to Training programme (SuppoRTT) was developed.

We at Sheffield Health and Social Care NHS Foundation Trust devised a unique “Mindful SuppoRTT” initiative and were successful in securing funding from HEE. Part of which was the organisation of a conference aimed at various groups of doctors including those who have previously had time out of training, are currently out of training and those considering time out.

The Sheffield Mindful SuppoRTT Programme not only aimed to provide a structured and systematic process for planning and returning from absence, but also focussed on enhancing performance through promoting the wellbeing of participants and supporting them with important clinical and non-clinical skills.

**Method:**

2-day twice yearly conferences, which covered training on speciality specific as well as non-technical skills were organised. The clinical workshops covered interactive sessions of common and emergency clinical scenarios. A wide range of non-technical skills such as an introduction to mindfulness, tai chi, resilience, team-working and leadership, “Thinking Environment” and meditation were introduced and developed using bespoke training. Feedback was collected at the end of each conference day. The attendees were asked to use a 5-point Likert scale (5 being the highest) to rate their satisfaction with the day and to highlight which sessions they found most and least useful.

**Result:**

The attendee satisfaction rate was high. The first conference had ratings of 56% of attendees scoring 5 (excellent) and the remainder scoring 4 (very good). The second conference achieved even higher satisfaction ratings with 94% of attendees scoring 5 and the remainder scoring 4.

**Conclusion:**

The conference had high attendee satisfaction. The hope is to expand on its success and open it up to delegates from all specialities within HEE South Yorkshire and the Humber. Evaluation of the long-term impact of this programme is also warranted.

